# Combined Ascorbic Acid and Mild Heat Treatment to Improve the Quality of Fresh-Cut Carrots

**DOI:** 10.3390/foods13121904

**Published:** 2024-06-17

**Authors:** Sen Ma, Ning Zhou, Yinghua Fu, Jiayi Wang

**Affiliations:** National Demonstration Center for Experimental Biology Education, Xinjiang Key Laboratory of Biological Resources and Genetic Engineering, College of Life Science and Technology, Xinjiang University, Urumqi 830046, China

**Keywords:** fresh-cut carrots, minimal processing, hurdle technology, pathogen

## Abstract

Mild heat (MH) treatment and ascorbic acid (AsA) addition can improve the quality of fresh-cut produce when used individually; however, their combined effect remains unclear. Herein, fresh-cut carrots were used as models to explore the effects of MH (50 °C)–AsA (0.5%) on quality properties including reactive oxygen species (ROS) metabolism, antioxidants, lignin metabolism, naturally present microbes, and inoculated pathogens (*Escherichia coli* O157: H7 and *Salmonella* Typhimurium) during storage (0–5 d, 4 °C). The results indicate that the antioxidant properties in the MH–AsA group were consistent with those of single treatments, resulting in a consistent ROS-scavenging effect. From day 3–5, lignin synthesis was significantly inhibited by MH–AsA as compared with single treatments, probably because the two enzymes (phenylalanine ammonia-lyase and peroxidase) responsible for lignin synthesis exhibited lower expressions. Microbial analysis revealed that MH–AsA treatment led to the lowest counts of both pathogens and aerobic mesophilic bacteria at 0–5 d. Conversely, the inhibitory effect of MH–AsA treatment on mold and yeast was consistent with the single treatments. These results suggest that MH–AsA is a low-cost and safe approach to improve the physiological characteristics of fresh-cut produce while reducing microbial risk.

## 1. Introduction

Carrots are a root vegetable that is widely grown and consumed worldwide [1] and are popular owing to their rich nutritional value and unique flavor [2]. Although fresh-cut carrots are convenient and quick for consumers [3], the cutting process promotes microbial reproduction and lignification [4], thereby reducing their quality.

Mild heat (MH) treatment, which uses water at temperatures of 35–60 °C for 10 s to 1 h, is often employed to treat fruits and vegetables [5]. For example, Zhang et al. [6] reported that the MH (40 °C, 5 min) treatment of sliced mushrooms could improve antioxidant enzyme activity and antioxidant capacity while inhibiting reactive oxygen species (ROS) formation and browning-related enzyme activity. Moreover, the pre-storage treatment of fresh lotus roots at 40, 50, and 60 °C for 30 min could effectively prevent water loss, microbial growth, browning, and tissue softening [7]. Rodoni et al. [8] immersed peppers in water at 45 °C for 3 min, which reduced the incidence of soft rot and prevented drying out, weight loss, and color changes. Previous studies have reported that the MH (60 °C, 60 min) treatment of carrots maintained their hardness, improved their texture, and inhibited peroxidase (POD) activity [9].

Also known as vitamin C, ascorbic acid (AsA) possesses antioxidant properties and can inhibit the enzymatic browning of fruits and vegetables, reduce o-quinone content, and slow the oxidative deterioration of food [10]. Polyphenol oxidase (PPO) is also associated with enzymatic browning. Studies have shown that AsA treatment can inhibit PPO activity in bean sprouts [11], lotus root slices [12], and strawberries [13] to reduce enzymatic browning, thereby prolonging the storage period. In addition, AsA treatment can delay the wound healing process in fresh-cut potatoes via ROS production, antioxidant enzyme activity enhancement, and the cyclic metabolic activity of ascorbate–glutathione. This phenomenon improves the overall antioxidant capacity, balances the production of ROS and antioxidant defense systems, inhibits browning, and prolongs shelf life [14]. Jalili et al. [15] investigated the effect of AsA treatment on the quality of grapes stored at −3 °C. This treatment increased the activities of catalase, ascorbate peroxidase, and superoxide dismutase in grapes, thereby reducing ROS content and damage due to freezing.

These two technologies have been combined and applied to preserve and improve the quality of fresh-cut fruits and vegetables. For example, Aguayo et al. [16] combined MH (48 °C, 2 min) with calcium ascorbate (6%). This combined treatment maintained the quality of fresh-cut apples, particularly their sensory flavor and aroma, and extended their shelf life from 14 to 21 d. Goyeneche et al. [17] used AsA (2%, 5 min) and MH (50 °C, 1.5 min) to treat radish slices, thereby prolonging their shelf life by reducing the population of microorganisms. Although the application of MH [9] and AsA [18] to fresh-cut carrots has been reported, few studies have investigated whether a combination of the two can produce a superimposed effect. Therefore, this study explored the effects of MH combined with AsA on the quality of fresh-cut carrots by analyzing their physiological and microbiological indicators.

## 2. Materials and Methods

### 2.1. Sample Preparation

Carrots were purchased from the local market, stored at 4 °C, and used within 5 days. On the day of the experiment, samples with no mechanical damage, rot, or deterioration were carefully selected. The samples were rinsed thoroughly with tap water to remove any dirt. Thereafter, they were peeled and cut into cubes in a dicer (MDQ20, MUPOOL, Foshan, China; 8 mm × 8 mm × 8 mm).

### 2.2. Pathogen Inoculation

*Escherichia coli* O157:H7 (ATCC700728) and *Salmonella* Typhimurium (ATCC14028) recommended for fresh produce inoculation [19] were selected in this study. A single colony of the pathogen was transferred into nutrient broth (Hopebio, Qingdao, China), and the culture was incubated overnight with shaking at 37 °C. The cell pellet was obtained by centrifugation at 12,000× *g* for 10 min at 4 °C. After washing three times with 0.85% NaCl, the pathogen was resuspended in 0.85% NaCl to prepare an inoculation solution (10^9^ CFU/mL). The sample was immersed in the bacterial suspension for 10 min with a ratio of 1:9 (*w*/*v*). Subsequently, the inoculated sample was placed in a biosafety cabinet and the fan was opened for natural air-drying. Then, the sample was placed in a sterilized dish and stored at 4 °C for 12 h to allow sufficient bacterial attachment.

### 2.3. Sample Processing

In this experiment, the experimental conditions were optimized through preliminary experiments. The optimal processing parameters, including time, temperature, and Vc concentration, were determined to be 5 min, 50 °C, and 0.5%, respectively. The ratio of carrots to the treatment solution was 1:5 (*w*/*v*). Samples (150 g) were packed in preservation boxes (133 × 133 × 21 mm), sealed with plastic wrap (Heng Na Kang Wrap), and stored at 4 °C for 5 days. Samples were collected on days 0, 1, 3, and 5 and processed in an IKA liquid nitrogen grinder to obtain powders for subsequent analysis. The untreated sample served as the control.

### 2.4. Determination of Reactive Oxygen Species (ROS) and Malondialdehyde Levels

The quantification of hydrogen peroxide (H_2_O_2_), malondialdehyde (MDA), and the rates of superoxide anion (O_2_^−^) production was performed using specific kits obtained from the manufacturer (Gerace Bio, Suzhou, China).

### 2.5. Antioxidant Enzyme Activity and Ascorbic Acid (AsA) Determination

The levels of superoxide dismutase (SOD), catalase (CAT), ascorbate peroxidase (APX), and ascorbic acid (AsA) were determined using the respective kits obtained from the manufacturer (Gerace Bio, Suzhou, China).

### 2.6. Lignin Synthesis and Phenolic Content Determination

The activities of phenylalanine ammonia-lyase (PAL), peroxidase (POD), cinnamyl-alcohol dehydrogenase (CAD), and lignin were determined using the respective kits obtained from the manufacturer (Gerace Bio, Suzhou, China). The phenolic content was determined following the protocol outlined by Wang et al. [19].

### 2.7. Microbiological Analysis

On the day of sampling, the sample was mixed with 0.85% NaCl at a ratio of 1:9 (*w*/*v*) and agitated for 2 min at 120 rpm to prepare the suspension. The serially diluted suspension (1 mL) was pour-plated onto plate count agar (Hopebio, Qingdao, China) and incubated for 2 days at 37 °C to quantify the aerobic mesophilic count (AMC). For molds and yeasts (M&Y), 1 mL of the diluted suspension was pour-plated onto Rose Bengal chloramphenicol agar (Hopebio, Qingdao, China) and incubated for 5 days at 28 °C. For pathogens, 1 mL of the diluted suspension was spread-plated onto modified sorbitol MacConkey agar (Hopebio) and xylose-lysine-deoxycholate agar (XLD; Hopebio, Qingdao, China) for the analysis of *E. coli* O157:H7 and *S.* Typhimurium, respectively. The plates were incubated for 24 h at 37 °C.

### 2.8. Statistical Analysis

Each experiment was performed in triplicate. The experimental data were analyzed using the SPSS v.26 (SPSS Inc., Chicago, IL, USA) software through univariate analysis, and significance testing was performed using Duncan’s multiple comparison. Differences were considered significant at *p* < 0.05.

## 3. Results and Discussion

### 3.1. Effect of Different Treatments on the O_2_^−^, H_2_O_2_, and Malondialdehyde (MDA) Contents of Fresh-Cut Carrots during Storage

**Figure 1:**
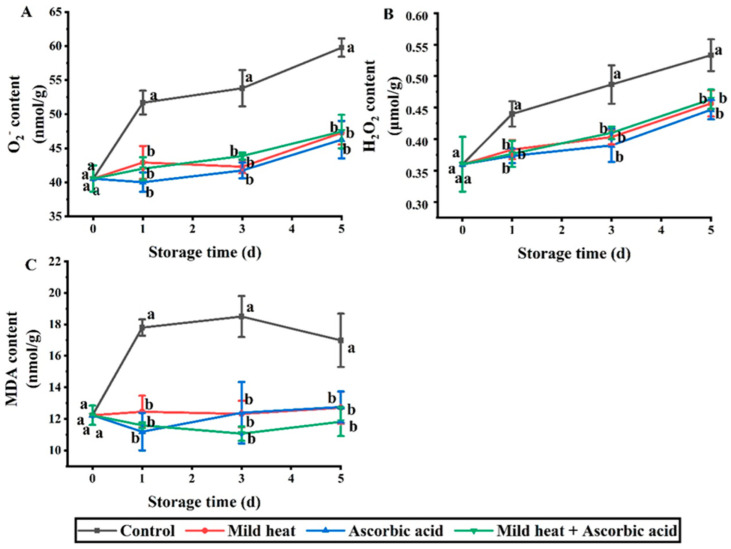
Effects of different treatments on O_2_^−^ (**A**), H_2_O_2_ (**B**), and MDA (**C**) contents of fresh-cut carrots during storage. Different lowercase letters in the same day indicate significant differences (*p* < 0.05).

Fresh-cut produce is subjected to external stresses that result in increased O_2_^−^ and H_2_O_2_ contents [20]. In high concentrations, these molecules can impede macromolecules, such as proteins, lipids, and nucleic acids, thereby causing cell necrosis [21]. These changes negatively affect the quality of fresh-cut produce. As shown in Figure 1A,B, the concentrations of O_2_^−^ and H_2_O_2_ increased from 0 to 5 d. When plants are subjected to external stresses, such as cutting, their respiratory burst oxidase homologs (RBOHs) are activated. RBOHs transfer electrons from cytoplasmic nicotinamide adenine dinucleotide phosphate molecules to outside of the cytoplasm to produce O_2_^−^, which is then converted to H_2_O_2_ under the action of superoxide dismutase (SOD) [22,23]. The concentrations of O_2_^−^ and H_2_O_2_ in the control group were significantly higher than those in the treatment groups on days 1–5 (*p* < 0.05); however, no significant differences were observed between the different treatment groups (*p* > 0.05). Therefore, the three different treatments inhibited the production and metabolism of O_2_^−^ and H_2_O_2_ in fresh-cut carrots, with no significant differences among them.

MDA is the primary product of membrane lipid peroxidation, and its content indicates the intensity and rate of lipid peroxidation, thereby reflecting the effective index of cellular oxidative damage in plants following an adverse injury [24,25]. Moreover, MDA interacts with proteins and nucleic acids and disrupts cell membrane fluidity [26]. As shown in Figure 1C, the MDA content in the control group increased at 0–1 d and attained a maximum value of 18.50 nmol/g on day 3. These results indicated that the carrots produced numerous O_2_^−^ molecules following external stress, which resulted in membrane lipid peroxidation. Conversely, the MDA contents in the MH, AsA, and MH–AsA treatment groups were 12.32, 12.39, and 11.06 nmol/g, respectively. The MDA content was significantly higher in the control group than in the treatment groups on days 1–5; however, no significant difference was observed among the three treatment groups.

### 3.2. Effect of Treatment Methods on Antioxidant Enzymes and the AsA Activity of Fresh-Cut Carrots during Storage

**Figure 2:**
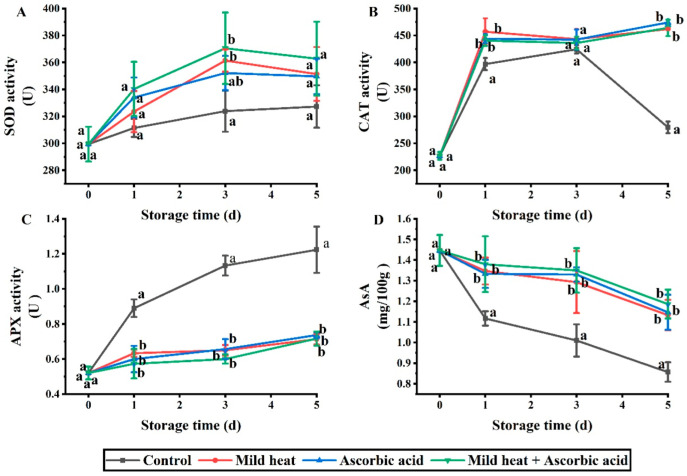
Effects of different treatments on SOD (**A**), CAT (**B**), APX (**C**), and AsA (**D**) activities of fresh-cut carrots during storage. Different lowercase letters in the same day indicate significant differences (*p* < 0.05).

Antioxidant enzymes promote the antioxidant scavenging of free radicals [27], and SOD and catalase (CAT) are the key antioxidant enzymes that scavenge ROS [28]. SOD is an important endogenous antioxidant enzyme in cells and an integral part of the first line of defense against ROS [29]. For example, when the O_2_^−^ content increases, the activity of SOD increases to protect cells from oxidative stress by degrading O_2_^−^ to H_2_O_2_, followed by the breakdown of H_2_O_2_ by CAT into harmless H_2_O and O_2_ [28]. After cutting, the ROS content increased and accumulated; thus, SOD and CAT activities increased to restore the ROS content to normal levels. As shown in Figure 2A, the SOD activity of fresh-cut carrots in the control group increased from 0 to 5 days. On day 3, SOD activity attained maximum values of 361.43, 352.16, and 370.55 U/g for the MH, AsA, and MH–AsA treatment groups, respectively. Although the SOD activity of the treatment groups was higher than that of the control group within 1–5 d, only on the third day were the MH and MH–AsA treatment groups significantly higher than those of the control group (*p* < 0.05). As shown in Figure 2B, the CAT activity of the control group increased from 0 to 3 d and decreased from 3 to 5 d. Conversely, the CAT activity of the treatment groups was significantly higher than that in the control group on days 1 and 5 (*p* < 0.05). Therefore, the three treatments promoted the activities of SOD and CAT, thereby reducing ROS content; however, no significant differences were observed between the different treatment groups on days 1–5 (*p* > 0.05).

Ascorbate peroxidase (APX) plays a key role in maintaining intracellular ROS level homeostasis by catalyzing the conversion of H_2_O_2_ to O_2_^−^ and H_2_O. This process alleviates the damage caused by H_2_O_2_ to plant cells and protects them from oxidative stress [30]. As shown in Figure 2C, the APX activity in the control group on days 1–5 was significantly higher than that in the treatment groups; however, no significant differences were observed among the different treatment groups. This may be because ROS levels were lowered by CAT and SOD activities in the treatment groups, and low levels of ROS could not induce the expression of APX.

As an antioxidant, AsA content decreases with age in fresh produce [31]. As shown in Figure 2D, the AsA content in the treatment groups decreased from 0 to 5 d, whereas that in the control group exhibited a greater decrease. Thus, the AsA content in the control group was significantly lower than those in the treatment groups on days 1–5, with no significant difference between them. Begara-Morales et al. [32] observed that APX activity increased in pea leaves when H_2_O_2_ content increased under salt stress. Moreover, AsA can be oxidized to MDHA by APX in plants; therefore, AsA content is negatively correlated with APX activity [33]. In this study, the content of H_2_O_2_ increased on days 0–5, which promoted APX expression, thus confirming a negative correlation between APX and AsA.

### 3.3. Effect of Treatment Methods on the Lignin Synthesis of Fresh-Cut Carrots during Storage

**Figure 3:**
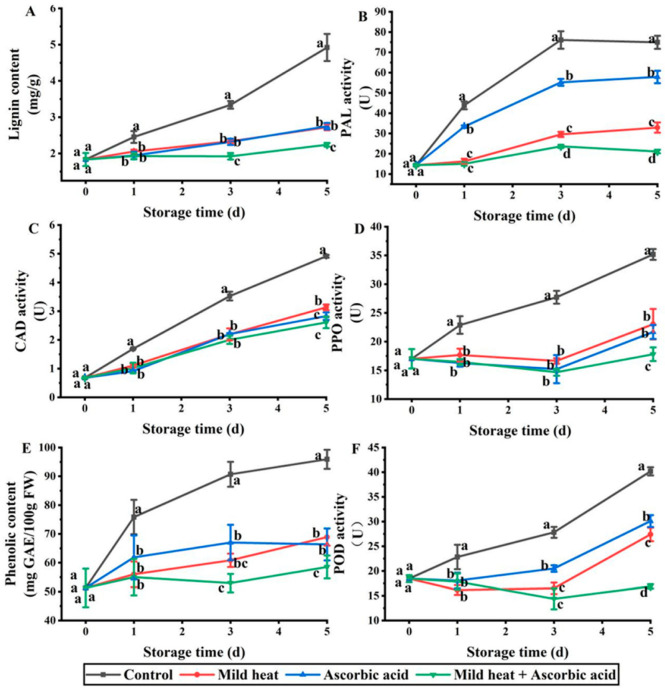
Effects of different treatments on lignin (**A**) content, PAL (**B**), CAD (**C**), PPO (**D**) activity, phenol content (**E**), and POD (**F**) activity of fresh-cut carrots during storage. Different lowercase letters in the same day indicate significant differences (*p* < 0.05).

Lignin is the primary structural component of cell walls, and its biosynthesis is controlled by external stimuli such as light, wounds, and pathogen infections [34]. Therefore, cutting promotes lignin synthesis. In the present study, the lignin content in the control group exhibited an upward trend during storage; however, the lignin synthesis rate was significantly reduced after all three treatments (Figure 3A). On days 1–5, the MH and AsA treatment groups had the same effect on lignin; however, from the third day, the lignin content in the MH–AsA treatment group was significantly lower than that in the single treatments.

The precursors of lignin biosynthesis are produced via phenylpropane metabolism. This pathway begins with the deamination of phenylalanine to form cinnamic acid, which then undergoes a series of hydroxylation, methylation, and reduction reactions to produce the monomers required for lignin biosynthesis [35]. Phenylalanine ammonia-lyase (PAL) is the enzyme that participates first in lignin biosynthesis [36]. Thus, PAL activity was significantly inhibited by the three treatments on days 1–5 d, with the lowest PAL activity observed in the combination treatment group (Figure 3B). CAD is the last enzyme to perform in the lignin synthesis pathway and is responsible for catalyzing hydroxycinnamaldehyde to form alcohol [37]. In the present study, CAD activity increased during storage (Figure 3C) and was significantly lower in the three treatment groups than in the control group. No significant differences were observed between the three treatment groups on days 1–3. However, the CAD activity in the AsA and MH–AsA treatment groups was significantly lower than that in the MH treatment group on day 5.

PPO is responsible for the formation of quinones by catalyzing polyphenols, and its activity is negatively correlated with the polyphenol content [38]. PPO activity in the three treatment groups was significantly lower than that in the control group on days 1–5 (Figure 3D), and its activity in the combination treatment group was significantly lower than that in the single treatment groups on day 5. PAL is a key enzyme in phenolic synthesis and is positively associated with phenolic compounds [39]. In this study, the phenolic content in the three treatment groups was lower than the control group on days 1–5 (Figure 3E). This result was consistent with that for PPO, indicating that the lower polyphenol content in the three treatment groups compared to that of the control group was due to the inhibition of PAL activity by the three treatments.

POD catalyzes the formation of lignin monomers to form lignin [40], and the activities of PAL and POD are positively associated with lignin content [41]. POD activity in the combination treatment group was lower than that in the single treatment groups on days 3–5 (Figure 3F). The lowest phenolic content was observed in the combination treatment group on days 3–5, which was consistent with the trend observed for lignin and PAL. This result indicated that MH–AsA treatment hindered phenolic and lignin synthesis by inhibiting PAL and POD activities, thereby resulting in the lowest lignin content on days 3–5.

### 3.4. Inactivation Efficacy of AsA, MH, and MH–AsA Treatment against Naturally Present Microbes and Food-Borne Pathogens

**Figure 4:**
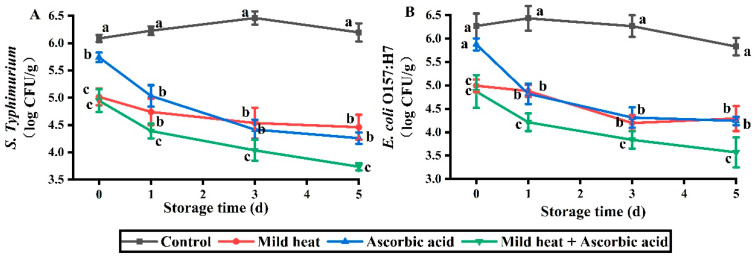
Effects of different treatments on *S.* Typhimurium (**A**) and *E. coli* O157:H7 (**B**) contents of fresh-cut carrots during storage. Different lowercase letters in the same day indicate significant differences (*p* < 0.05).

*Salmonella* is the most common contaminating pathogen in fresh-cut produce and accounts for 48% of cases, followed by *E. coli* O157:H7 [42]. MH is the preferred physical method for combination with other methods to develop hurdle technologies [43], while natural products are favored by consumers. Thus, previous studies have successfully combined MH treatment with natural products to inactivate food pathogens. For example, additional count reductions of *Cronobacter sakazakii*, *Staphylococcus aureus*, *E. coli* O157:H7, and *E. coli* were achieved with the combinations MH–*Dryopteris erythrosora*, MH–citral, MH–carvacrol, MH–caprylic acid, and MH–vanillin, compared to those of single treatments [44,45,46,47]. However, our understanding of the inactivation efficacy of MH–AsA treatment against pathogens in fresh-cut produce is limited. Therefore, the inactivation efficacy of *S.* Typhimurium and *E. coli* O157:H7 on fresh-cut carrots was analyzed. The results revealed that *S.* Typhimurium counts were 5.02, 5.74, and 4.95 log CFU/g following treatment with MH, AsA, and MH–AsA, respectively, which were significantly lower than those of the control group (Figure 4A). During storage (1–5 d), *S.* Typhimurium showed a decreasing trend in the three treatment groups. Moreover, the efficacy of the combination treatment was significantly higher than that of the single treatments. When comparing AsA with MH treatment, consistent efficacy was observed on days 1–5. The counts of *E. coli* O157:H7 in the MH and MH–AsA treatment groups were 4.99 and 4.87 log CFU/g, respectively, on day 0, which were significantly lower than those in the control group (Figure 4B). During storage (1–5 d), the efficacy of MH treatment against *E. coli* O157:H7 was consistent with that of AsA treatment. Similar to the results observed for *S.* Typhimurium, MH–AsA treatment resulted in the lowest counts of *E. coli* O157:H7 during storage.

In general, hurdle technology combines different disinfection methods with different antibacterial mechanisms to lead additional count reductions as compared with single treatment [43]. As the main food additives, organic acids (e.g., citric acid, malic acid, and lactic acid) are generally used to adjust food flavor and serve as food preservatives in the food industry [48]. After attaching to the cell membrane, the acid molecules penetrate the cell and dissociate into hydrogen ions and acid radicals. The hydrogen ions increase osmotic pressure, leading to ATP over-consumption; acid radicals block DNA and RNA synthesis, ultimately leading to cell death [48]. Similarly, the antibacterial mechanism of AsA is to induce intracellular damage [49]. For MH, the antibacterial mechanism is to damage the cell membrane and induce protein denaturation [44]. Thus, AsA and MH inactivate pathogens through different mechanisms, explaining the additional count reductions achieved by AsA–MH. At day 0, the inactivation capacity of AsA against two pathogens was significantly lower than MH, due to the weak antibacterial activity of AsA. Similar results were observed by Hismiogullari et al. [50]; they found that the cytotoxic activity of AsA against mammalian cells was significantly lower than lactic acid (LA) and acetic acid (AA). We also observed that *E. coli* O157:H7 was not significantly inactivated by AsA at day 0, in contrast with the observations on *S.* Typhimurium. This result may be due to the different cell structure between these two pathogens. The study of Hyun et al. [51] employed AsA to disinfect *E. coli* O157:H7 present on tomatoes and also found ineffective results. However, AsA has shown strong antibacterial activity during food storage. *Salmonella* in cheese was significantly controlled by AsA during storage, without affecting the sensory and nutritional properties [52]. Hatano et al. [53] found that AsA simultaneously improved the antioxidant and antibacterial activity of epigallocatechin solution. At the end of storage, *E. coli* O157:H7 present on AsA-coated tomatoes was undetectable, whereas the count reductions achieved by carvacrol and citric acid were 2.01 and 1.46 log CFU/g, respectively [51].

**Figure 5:**
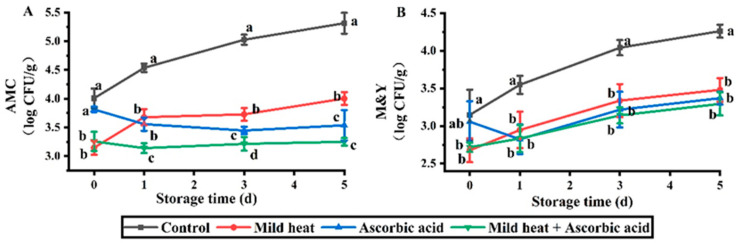
Effects of different treatments on counts of AMC (**A**) and M&Y (**B**) of fresh-cut carrots during storage. Different lowercase letters in the same day indicate significant differences (*p* < 0.05).

For naturally occurring microbes, AMC and M&Y were significantly inactivated by MH and MH–AsA treatments on day 0 and significantly inactivated by AsA treatment during storage (Figure 5). The lowest AMC values were observed in the MH–AsA treatment group on days 1–5, whereas its efficacy against M&Y was consistent with the two single treatments. This result may be due to the stronger cell membrane structure of fungi than that of bacteria, leading to the stronger resistance of fungi [54,55]. In a previous study, the M&Y present in cherry tomatoes were not further inactivated by ultrasound–peracetic acid treatment compared to single treatments [42]. Moreover, the M&Y on lettuce were not significantly inactivated by an increasing aqueous ozone concentration from 0.5 to 1.0 ppm [56].

From the perspective of microbial ecology, a large number of microbial species were excited on the produce surface, and co-exclusion and co-occurrence interactions were observed between each microbe [54]. As an external stimulus, disinfection can disturb ecological balance to promote bacterial growth. In a previous study, Wang et al. [54] found that *Xanthomonas*, a plant pathogen, was the dominant genera on lettuce surfaces, and its relative abundance was significantly increased from 24.73% to 47.53% after washing with propionic acid. Another study employed an AA, LA, and acid mixture (0.8%AA + 0.2%LA) to disinfect fresh-cut lettuce and found that the acid mixture and individual acids significantly decreased the abundance of *Massilia* spp. and *Alkanindiges* spp., but there was a significant increase in *Escherichia-Shigella* abundance (LA: 0.003–58.82%; AA: 0.01–55.34%; acid mixture: undetected to 50.71%) [57]. The author concluded that acid disinfection may have altered the microbial ecology on the produce surface, stimulating bacteria growth such as that of *Escherichia-Shigella*. Thus, hurdle technology was proposed to maintain a stable microbial composition on fresh produce. For example, a recent study combined ultrasound and aqueous ozone to wash fresh-cut lettuce and found that the relative abundance of *Escherichia-Shigella* in the combination group was significantly lower than in the single treatment groups [56]. In this work, we combined AsA with MH to disinfect fresh-cut carrots and found that AsA–MH showed a superior capacity in controlling AMC as compared with AsA and MH.

## 4. Conclusions

Fresh-cut carrots were selected as models to investigate a low-cost and highly safe hurdle technology, which comprised a combination of MH and AsA treatments. In particular, changes in the physiological properties and microbial counts of fresh-cut carrots were analyzed. The primary findings were as follows: (1) The combined treatment had the same effects on the antioxidant properties and ROS-scavenging activity of fresh-cut carrots as those of the single treatments. (2) Compared with the single treatments, the combined treatment achieved a significant inhibitory effect on lignin synthesis on days 3–5, which was attributed to PAL and POD inactivation. (3) The combined treatment resulted in the lowest AMC, *S.* Typhimurium, and *E. coli* O157:H7 counts during storage. These results provide a potential hurdle technology for improving the quality of freshly cut products. Although the activities of lignin metabolism enzymes (POD, CAD, and PAL) were analyzed in this study, an in-depth exploration of metabolites and gene changes in the lignin metabolism pathway remains lacking and should be further analyzed using omics technology.

## Data Availability

The original contributions presented in the study are included in the article, further inquiries can be directed to the corresponding author.
